# Intermuscular Adipose Tissue Is Muscle Specific and Associated with Poor Functional Performance

**DOI:** 10.1155/2012/172957

**Published:** 2012-05-14

**Authors:** Lori J. Tuttle, David R. Sinacore, Michael J. Mueller

**Affiliations:** ^1^Department of Orthopaedic Surgery, University of California, San Diego, Mail Code 0863, La Jolla, CA 92093, USA; ^2^Program in Physical Therapy, Washington University in St. Louis, MO 63108, USA

## Abstract

*Purpose*. People with obesity, diabetes, and peripheral neuropathy have high levels of intermuscular adipose tissue (IMAT) volume which has been inversely related to physical function. We determined if IMAT is muscle specific, if calf IMAT is different between a healthy obese group (HO), a group with diabetes mellitus (D), and a group with diabetes mellitus and peripheral neuropathy (DN), and if IMAT volume or the ratio of IMAT/muscle volume is related to physical function in these groups. *Methods*. 10 healthy obese people, 11 with type 2 diabetes, 24 with diabetes and peripheral neuropathy, had assessments of muscle morphology, physical function and muscle performance. *Results*. The gastrocnemius muscle had a higher ratio of IMAT/muscle volume than any other muscle or compartment. There were no differences between groups in calf muscle or IMAT volumes. Calf IMAT was inversely related to physical performance on the 6-minute walk test (*r* = −0.47) and physical performance test (*r* = −0.36). IMAT/muscle volume was inversely related to physical performance (PPT, *r* = −0.44; 6 MW *r* = −0.48; stair power, *r* = −0.30). *Conclusions*. IMAT accumulation varies in calf muscles, is highest in the gastrocnemius muscle, and is associated with poor physical performance.

## 1. Introduction

 Previous research has shown that people with obesity, diabetes, and peripheral neuropathy have significantly greater amounts of intermuscular adipose tissue (IMAT) in the calf compared to a nonobese control group and that this calf IMAT was associated with poor physical performance [1]. The unique contributions of obesity, diabetes, and diabetes in combination with peripheral neuropathy to the amount of IMAT in skeletal muscle, however, are not clear. In addition, the relationship between calf IMAT and physical performance is not clear in these groups. IMAT is defined as the visible adipose tissue beneath muscle fascia and between muscle groups [2, 3]. IMAT in the thigh has been linked to insulin resistance and has been described as a unique adipose tissue depot that is similar to visceral adipose tissue in its risks for metabolic impairment [4–6], but little is known about IMAT in the calf muscles. Previous investigators [5, 6] have shown that IMAT is linked to insulin resistance and that denervation [7] can also contribute to fat infiltration in muscle, but it is unclear how the combination of obesity, diabetes, and peripheral neuropathy impacts IMAT infiltration.

 It is also unknown whether calf IMAT accumulation is muscle or muscle compartment specific, that is, whether one muscle or a group of muscles tends to have more IMAT than others. Identifying preferential differences in IMAT accumulation may help to understand the purpose of IMAT. Muscles in the calf have different distributions of fast twitch and slow twitch fibers. For example, the gastrocnemius muscle is considered predominantly a fast twitch muscle and is used more for large force production, while the soleus muscle is considered more slow twitch and is a postural muscle that is better suited for using lipids as a fuel source [8]. It has been shown in animal models that there is a difference in fatty acid transport in muscles with different fiber type distributions (type I/red muscles are more oxidative than type II/white) and that fatty acid transport and triacylglycerides in muscle are impacted by insulin resistance and diabetes [9]. Additionally, increased triglyceride storage has been shown in the type I fibers of the soleus in obese rats [10]. It is unknown in humans whether muscles with different “predominance” of fiber types are targeted by IMAT infiltration in the presence of obesity, diabetes, and diabetes combined with neuropathy. A better understanding of IMAT distribution within the calf will provide us with insight into muscles that may be more compromised by fatty infiltration and may lead to improved rehabilitation strategies in these populations.

 Therefore, the purposes of this study were to determine if IMAT accumulation is muscle specific, that is, determine difference in IMAT volumes between individual muscles and muscle compartments, if calf IMAT is different between groups of healthy obese people (HO), a group with diabetes mellitus (D), and a group with diabetes and peripheral neuropathy (DN), if IMAT and/or the ratio of IMAT/muscle volume is related to function in these groups. We hypothesized that the soleus muscle would display more IMAT than the gastrocnemius muscle and all other calf compartments across the three groups due to the predominance of slow twitch fibers and higher lipid oxidation capacity in the soleus muscle. Additionally, we hypothesized that the D and DN groups would have greater volumes of IMAT in the calf, and similar calf muscle volumes compared to the HO group, and that the DN group would display the largest volume of calf IMAT of all 3 groups. We hypothesized that calf IMAT volume would be inversely correlated with measures of physical performance.

## 2. Methods and Procedures

### 2.1. Participants

 Forty-five subjects participated in this study (Table 1). The groups were matched for age and BMI. Initially, there was an analysis of a group of 10 DN subjects who best matched the 10 individuals in the other 2 groups. However, this did not change the results and increased the DN group variability. This led to reporting the larger group of DN subjects.

Participants were recruited from the Washington University School of Medicine Diabetes Clinic, Washington University's Volunteers for Health, the Center for Community Based Research, and from diabetes clinics in the surrounding St. Louis community. This study is part of a larger study investigating the effect of exercise for people with diabetes and peripheral neuropathy. Participant characteristics are listed in Table 1. Participants were excluded if they weighed more than 300 pounds (equipment weight limit) or had a history of severe foot deformity or amputation, any co-morbidity or medications that would interfere with exercise (such as severe rheumatoid arthritis, peripheral arterial disease (absent pulses), dialysis, or current cancer treatment). Participants provided written informed consent. This study was approved by the Human Research Protection Office at Washington University in St. Louis.

## 3. Assessments

### 3.1. Peripheral Neuropathy

Presence of peripheral neuropathy was determined based on both an inability to feel the 5.07 Semmes-Weinstein monofilament on at least one point on the plantar surface of the foot and on a vibration perception threshold greater than 25 V as measured with a biothesiometer applied to the plantar surface of the great toe [11, 12]. All subjects were tested for presence of neuropathy; to be included in the D and HO groups, subjects had to be able to feel the 5.07 monofilament and have a vibration perception threshold below 25 V.

### 3.2. Intermuscular Adipose Tissue (IMAT)

**Figure 1:**
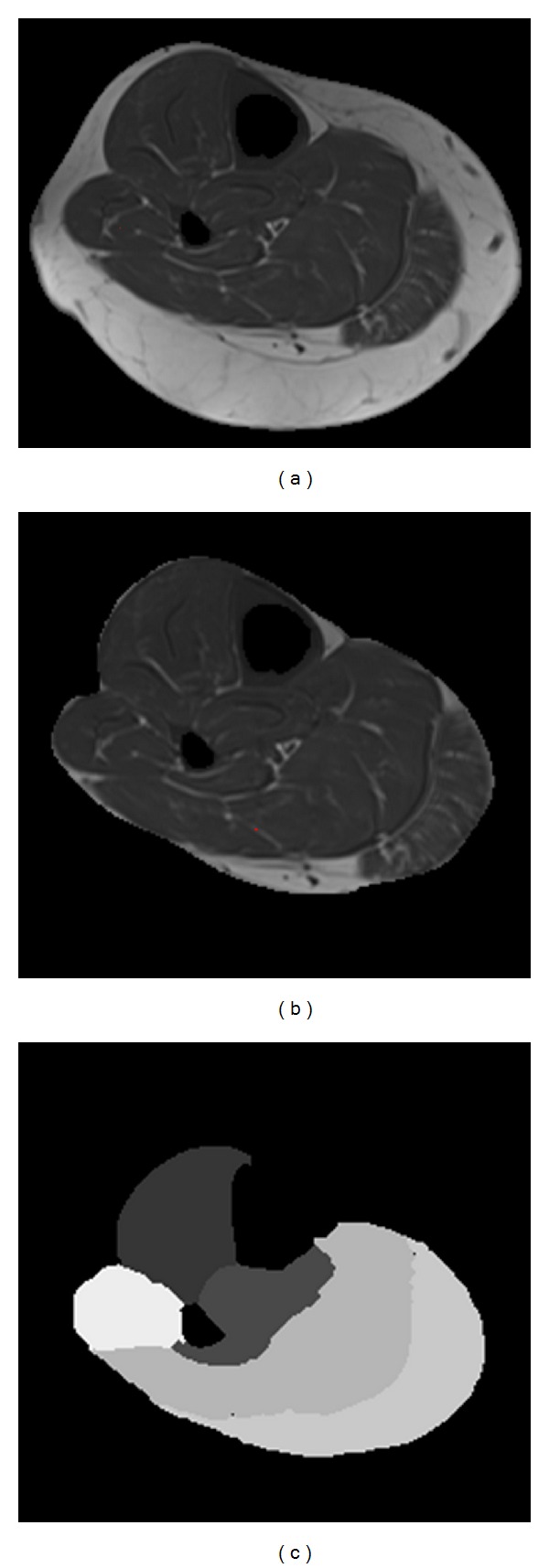
(a) MRI image of calf with bone removed. (b) Subcutaneous adipose tissue removed. (c) Calf divided into 5 compartments (anterior, lateral, deep, gastrocnemius, and soleus).

 Calf intermuscular adipose tissue volumes were quantified using MRI on the right leg of each participant. The MRI scans were performed with the participant in a supine position with a Siemens CP extremity coil placed over the right calf muscle. The MRI measurements were performed with a 3.0 Tessla superconducting magnet with a pulse sequence of TE = 12 milliseconds, TR = 1,500 milliseconds, matrix = 256 × 256; both a fat-saturated and a non-fat-saturated image were collected [1]. Thirty transverse slices were collected beginning at the joint space of the knee and proceeding distally. The slices were 7 mm thick with no interslice gap. Nine consecutive slices were selected to calculate muscle and IMAT volumes. Volumes were quantified using a PC workstation and custom Matlab software. The software uses voxel brightness to distinguish between muscle and adipose tissues [6, 13, 14]. The subcutaneous adipose tissue was removed from each image by drawing a line along the deep fascial plane surrounding the calf muscle so that only the fat within and between the muscles (IMAT) was remaining. The software uses edge detection algorithms to assist the user in separating the subcutaneous fat from the muscle as well as separating individual muscles and muscle compartments. In the calf, the muscle was divided into (1) the anterior compartment, (2) the lateral compartment, (3) the deep compartment, (4) the gastrocnemius muscle, and (5) the soleus muscle (Figure 1). Calf IMAT and calf muscle volumes are reported in cm^3^. An additional variable, IMAT per muscle volume, was also used in analysis. Based on test-retest reliability of 21 subjects (group included people who are obese, people with diabetes, and people with diabetes and peripheral neuropathy), the error in measuring muscle volume is less than 1% and less than 2% for measuring fat volumes on average in any muscle or compartment [15].

### 3.3. Six-Minute Walk Test

All participants performed the six-minute walk test [16] which was validated previously in obese adults [17]. The participants walked back and forth in a hallway between 2 cones that were placed 100 feet (30.5 m) apart. The participants were instructed that the goal was to walk as far as possible in 6 minutes. Six-minute walk distance was recorded as total distance walked (in meters).

### 3.4. Physical Performance Test (PPT)

The modified 9-item PPT was used to assess physical performance in all participants. This test is designed to mimic activities of daily living, and the 9-item PPT has been shown to correlate well with disability and frailty [18–20]. The 9-item PPT includes placing a book on a shelf, putting a lab coat on and taking it off, picking up a coin from the floor, a 25-foot walk down and back at a fast speed, turning in a 360-degree circle, simulated eating, writing a sentence, climbing a flight of stairs with 10 steps, and sitting to standing 5 times from a low chair. Each of the items is scored on a scale of 0–4 based on the time it takes to complete the task. Each task is performed twice, and the average time is used to determine the 0–4 score. A maximum score is indicated by a score of 36.

### 3.5. Stair Power Measure

 Stair power (in watts) was calculated based on the time it took each participant to climb a flight of 10 stairs as part of the PPT (average of 2 trials) using the following formula which was adapted from the stair sprinting power test [21]:

(1) $$\begin{array}{cc} \text{Stair}\;\text{Power} & =3.171\;*\;\text{Weight}\;\left(\text{kg}\right)/\text{time}\;\left(\text{sec}\right)\\ & \;\text{Climb}\;\text{avg}\;*\;\frac{1}{.1383}\;*\;\frac{1}{.7378},\\ \text{where}\;3.171 & =\;\text{distance}\;\text{traveled}\;\left(\text{m}\right),\\ \text{Climb}\;\text{avg} & =\text{average}\;\text{time}\;\text{to}\\ & \;\text{climb}\;\text{a}\;\text{flight}\;\text{of}\;10\;\text{stairs}\;\left(\text{sec}\right),\\ \frac{1}{.1383} & =\text{conversion}\;\left(\frac{\text{kgm}}{\text{s}}\;\text{to}\;\frac{\text{ftlbs}}{\text{s}}\right),\\ \frac{1}{.7378} & =\text{conversion}\;\left(\frac{\text{ftlbs}}{\text{s}}\;\text{to}\;\text{Watts}\right). \end{array}$$

Subjects were allowed to touch a handrail for balance, but not for pulling or pushing to ascend the stairs.

### 3.6. Ankle Dorsiflexion and Plantarflexion Peak Torque and Power

Concentric isokinetic ankle dorsiflexor and plantarflexor peak torque and power were assessed using a Biodex Multijoint System 3 Pro isokinetic dynamometer. The tests were performed at angular velocities of 60°/s. The average power at 60°/s was determined by the time-averaged integrated area under the curve at the constant velocity of movement in the available range of motion [1]. All participants were given 3 practice trials to ensure they were comfortable with the test. The mean values for peak torque and average power were calculated for 3 trials.

### 3.7. Statistical Methods

Statistical analyses were performed using Systat for windows, version 13.0. An analysis of variance was used to examine the main and interaction effects of calf IMAT and muscle volumes (gastrocnemius, soleus, anterior compartment, lateral compartment, and deep compartment), group (HO, D, DN), and measures of physical performance. Post hoc *t*-tests were used to examine differences in groups (HO, D, and DN) on the variables of calf IMAT volume, calf muscle volumes, and physical performance as needed based on the results of the ANOVA. A Pearson correlation was used to examine the associations between variables across all 45 subjects—all scatter plots were inspected and analyzed for outliers. Significance level was set at *P* = 0.05.

## 4. Results

 There were no group differences in age, BMI, or weight (*P* > 0.05). The HO group was significantly different from the other groups in HbA1c and DM duration—the D and DN groups were not significantly different on these measures. The gastrocnemius muscle had a higher ratio of IMAT/muscle volume than any other muscle or compartment (*P* = .005) across all participants (Table 2).

There were no group differences between any of the calf muscle or IMAT volume measures (Table 2). Group differences were determined for descriptive purposes and are contained in Table 3.

 Across all participants, calf IMAT volume was associated with BMI (*r* = 0.31) and IMAT volume was associated with poorer physical performance on the 6-minute walk test (*r* = −0.47) and the physical performance test (*r* = −0.36). IMAT/muscle volume was also associated with poor physical performance (PPT *r* = −0.44, 6 MW *r* = −0.48). Muscle volume was not strongly associated with 6-minute walk distance or physical performance test score but was associated with stair power (*r* = 0.51) (Table 4).

## 5. Discussion

This study is the first to report that the amount of IMAT/muscle volume in the calf is muscle and compartment specific in the pathologies of obesity, diabetes, and diabetes combined with peripheral neuropathy. The gastrocnemius muscle had the largest ratio of IMAT/muscle volume compared to any of the calf muscles and compartments, which was contrary to what we expected and to what has been reported in obese animal models [9, 10]. We speculate that perhaps those muscles with a predominance of fast-twitch fibers, such as the gastrocnemius muscle, are affected by IMAT accumulation preferentially or sequentially. The plantar flexor muscles are important for ankle stability, walking velocity, and cadence [22]. Furthermore, the gastrocnemius is used during powerful and phasic/burst type activity compared to the soleus muscle which is most active for postural control. Perhaps the gastrocnemius is more affected by IMAT than the soleus muscle due to a greater reduction in power activities compared to postural activities in these groups. Or, perhaps the reduced lipid metabolism in the gastrocnemius muscle compared to the soleus muscle results in greater IMAT storage rather than lipid oxidation. Additional studies are required to determine the underlying mechanisms for the IMAT accumulation in the gastrocnemius muscle and its propensity for having greater fat infiltration than other calf muscles. Understanding the muscle specific distribution of fat and the underlying mechanisms for fat infiltration may lead to enhanced treatment strategies to improve the health of the muscle and individual. For example, Marcus et al. [23] demonstrated that people with type 2 diabetes were able to improve performance, decrease fat, and increase lean tissue in the thigh muscles after a 16-week exercise program that included both aerobic and high-intensity eccentric exercise training. Perhaps specific rehabilitation strategies that target the gastrocnemius muscle could alter the fat infiltration and improve deficits in muscle performance and physical performance.

 Overall, the inverse correlation between calf IMAT volume and physical performance indicates that IMAT accumulation is associated with physical performance decline, but it appears that there are other factors, such as the presence of diabetes and/or neuropathy, that are key mediators of physical performance. The ratio of IMAT/muscle volume was inversely related to measures of muscle performance across all subjects. The ratio of calf IMAT/muscle volume may be an indicator of physical performance, but the IMAT/Muscle volume does not differ between those with diabetes and diabetes and neuropathy compared to a healthy obese group of subjects. These results are consistent with other reports in the literature and suggest measures other than absolute muscle volume or muscle cross-sectional area are needed to completely characterize calf muscle composition and muscle performance [1, 24] and suggest that perhaps IMAT/muscle volume may be an indicator of “muscle quality.” These data are also consistent with reports that people with D or DN have limitations in physical performance and function beyond what is fully explained by muscle changes alone [24, 25]. Certainly problems secondary to sensory neuropathy can contribute to these deficits in physical performance [26].

 We found, contrary to our expectations, that there were no group differences in measures of IMAT volumes or muscle volumes between a group with HO, a group with D, and a group with DN. These results indicate that diabetes and peripheral neuropathy were not associated with IMAT accumulation in the calf beyond their association with BMI in these groups of subjects. These results were surprising because our previous study indicated that a group with obesity, diabetes, and peripheral neuropathy had two times the volume of IMAT compared to a nonobese, nondiabetic, nonneuropathic control group [1]. Four of the six subjects with DN in that group were sampled from a patient sample with a history of foot ulcers rather than the community at large, so it is likely that we were capturing a group with more severe neuropathy in the previous study compared to what we report here.

 Of note, the HO group had an average HbA1c value of 5.8 which is indicative of people at risk for developing diabetes [27]. This HbA1c value is consistent with other reports in the literature that link IMAT with insulin resistance [5, 6], and this marginally high HbA1c value could be a potential indicator of those at risk for developing diabetes. Interestingly, the HO group had higher levels of physical performance than the D or DN groups, so perhaps an intervention targeted at minimizing IMAT could diminish risk for developing diabetes and mitigate the functional decline that is associated with diabetes and diabetes and peripheral neuropathy.

 There are limitations that should be considered. First, we have a relatively small sample size. Based on the small effect size between groups, a post hoc power analysis revealed that we would need to collect data on more than 3600 individuals to be powered to find group differences in total IMAT in the calf with a power of 0.80 and an alpha level at 0.05. The magnitude and impact of IMAT accumulation in specific calf muscles or compartments in people with severe diabetes and peripheral neuropathy requires additional investigation. We are limited in our ability to interpret results because we do not have biopsies or other biochemical measures of the individual muscles or adipose tissues to further elucidate characteristics beyond our macroscopic MRI measures. This study is also limited in that we do not have a measure of activity level for each participant, so it is possible that our groups could be different from each other in levels of activity. Future studies should characterize subjects on activity level, activity types (endurance versus strengthening exercise), and neuropathy severity to enhance interpretation of results. We do not have electrodiagnostic measures of neuropathy, and it possible that electrodiagnostic measures would have provided us with a more accurate measure of neuropathy severity including a measure of subclinical neuropathy in the D or HO groups. Since the group with DN was originally recruited for an exercise study, it is possible that we have a selection bias towards people with DN who are higher functioning. In addition, this group only had 2 people with a history of plantar foot ulcer, so we do not believe these results are generalizable to people with more severe complications and longer durations of diabetes and peripheral neuropathy. Lastly, the correlations between the different variables only indicate association and cannot determine cause and effect.

 In conclusion, this study found that increased calf IMAT volume accumulation was muscle specific; the gastrocnemius muscle had the largest ratio of IMAT/muscle volume of all of the calf muscles and compartments. In addition, calf IMAT was associated with poorer physical performance. The groups with D and DN had lower measures of physical performance than the HO group, suggesting that more severe impairment in metabolic pathology, along with IMAT accumulation, impacts physical performance.

## Figures and Tables

**Table 1:** Subject demographics by group. Values are means (SD).

Group	*N*	Gender (M/F)	Age (years)	BMI (kg/m^2^)	Weight (lbs)	Diabetes medication (oral only/insulin and oral)	HbA1c (%)	DM duration (years)
HO	10	4/6	64 (9)	32.9 (4.6)	213 (41)	NA	5.8 (0.2)	NA
D	11	5/6	56 (9)	35.5 (6.4)	226 (33)	6/5	8.1 (2.2)	8.1 (6.9)
DN	24	15/9	64 (13)	32.6 (6.3)	217 (45)	13/11	7.1 (1.3)	12.9 (9.0)

NA: not applicable.

**Table 2:** ANOVA results: muscle morphology measures; all values are means (SD) in cm^3^.

	HO	D	DN	*P* values
Muscle volume	404 (90)	434 (72)	407 (88)	0.65
IMAT volume	67 (54)	65 (36)	70 (40)	0.94
Anterior compartment muscle volume	62 (9)	69 (15)	65 (14)	0.57
Anterior compartment IMAT volume	7 (5)	10 (11)	9 (5)	0.53
Lateral compartment muscle volume	36 (10)	38 (9)	37 (11)	0.85
Lateral compartment IMAT volume	6 (5)	5 (3)	7 (4)	0.62
Deep compartment muscle volume	51 (15)	54 (13)	60 (12)	0.15
Deep compartment IMAT volume	9 (6)	8 (4)	11 (5)	0.23
Soleus muscle volume	127 (28)	130 (26)	118 (32)	0.46
Soleus IMAT volume	14 (14)	14 (8)	15 (10)	0.97
Gastroc. muscle volume	128 (31)	142 (27)	122 (40)	0.35
Gastroc. IMAT volume	31 (28)	27 (15)	28 (21)	0.90
IMAT/muscle volume	0.144 (0.07)	0.158 (0.11)	0.193 (0.16)	0.58

Anterior compartment: comprised of tibialis anterior, extensor digitorum longus, and extensor hallucis longus muscles.

Lateral compartment: comprised of peroneus longus and brevis muscles.

Deep compartment: comprised of the tibialis posterior, flexor digitorum longus, and flexor hallucis longus muscles.

**Table 3:** ANOVA results: physical performance measures. Values are means (SD).

	HO	D	DN	*P* values
DFPT (Nm)	5.2 (4.3)	15.3 (7.5)^a^	4.5 (5.3)	0.00
DFPOW (W)	2.3 (2.4)	9.8 (6.6)^a^	2.2 (3.2)	0.00
PFPT (Nm)	58.0 (18.6)	48.1 (13.0)	51.4 (16.5)	0.37
PFPOW (W)	45.9 (15.1)	38.7 (10.9)	41.9 (18.1)	0.38
6 MW (m)	512.4 (48)^a^	459.6 (80)	425.5 (98)	0.04
PPT	34 (1.5)	31 (2.4)	28 (4.0)	0.001*
Stair power (W)	808 (327)	671 (163)	601 (226)	0.04*

*Indicates all 3 groups are different.

^a^Indicates that the group is different from the other 2 groups.

DFPT: dorsiflexor peak torque; DFPOW: dorsiflexor power; PFPT: plantarflexor peak torque; PFPOW: plantarflexor power; 6 MW: six-minute walk distance; PPT: physical performance test (9 items).

**Table 4:** Correlation matrix.

	IMAT vol	6 MW	PPT	Stair POW	Muscle Vol	IMAT/Mus Vol
BMI	0.31*	−0.18	0.01	0.21	0.49*	0.08
IMAT vol		−0.47*	−0.36*	−0.18	−0.32*	0.93*
6 MW			0.79*	0.58*	0.25	−0.48*
PPT				0.60*	0.24	−0.44*
Stair Pow					0.51*	−0.30*
Muscle vol						−0.35*

*Indicates significance (*P* < 0.05).

IMAT: intermuscular adipose tissue volume; 6 MW: six-minute walk distance; PPT: physical performance test (9 items); Stair Pow: stair power; Muscle Vol: calf muscle volume; IMAT/MusVol: ratio of IMAT/muscle volume in the calf.
